# Enhancing brain tumor detection in MRI with a rotation invariant Vision Transformer

**DOI:** 10.3389/fninf.2024.1414925

**Published:** 2024-06-18

**Authors:** Palani Thanaraj Krishnan, Pradeep Krishnadoss, Mukund Khandelwal, Devansh Gupta, Anupoju Nihaal, T. Sunil Kumar

**Affiliations:** ^1^School of Computer Science and Engineering, Vellore Institute of Technology, Chennai, India; ^2^Department of Electrical Engineering, Mathematics and Science, University of Gävle, Gävle, Sweden

**Keywords:** brain tumor classification, Vision Transformers, rotational invariance, MRI, deep learning, rotated patch embeddings

## Abstract

**Background:**

The Rotation Invariant Vision Transformer (RViT) is a novel deep learning model tailored for brain tumor classification using MRI scans.

**Methods:**

RViT incorporates rotated patch embeddings to enhance the accuracy of brain tumor identification.

**Results:**

Evaluation on the Brain Tumor MRI Dataset from Kaggle demonstrates RViT's superior performance with sensitivity (1.0), specificity (0.975), F1-score (0.984), Matthew's Correlation Coefficient (MCC) (0.972), and an overall accuracy of 0.986.

**Conclusion:**

RViT outperforms the standard Vision Transformer model and several existing techniques, highlighting its efficacy in medical imaging. The study confirms that integrating rotational patch embeddings improves the model's capability to handle diverse orientations, a common challenge in tumor imaging. The specialized architecture and rotational invariance approach of RViT have the potential to enhance current methodologies for brain tumor detection and extend to other complex imaging tasks.

## 1 Introduction

The prevalence of brain tumors varies globally, with primary brain tumors representing 17% of all cancers and having an incidence of 39 per 100,000 person-years (Newton et al., 2022). In the United States alone, around 80,000 new primary brain tumors are diagnosed annually, with an approximate rate of 24 cases per 100,000 population (Reynoso-Noverón et al., 2021). Pediatric brain tumors, a significant cause of mortality in children, have an annual incidence of about 3 per 100,000 children (Abbas et al., 2021). The prevalence of brain tumors is influenced by factors such as age, gender, race, and region, with variations observed in different populations (Shobeiri et al., 2023). Wijethilake et al. (2021) in their paper explores the critical task of survival analysis in glioma patients, highlighting the integration of imaging and genetic data through advanced technologies to improve survival estimation. Furthermore, metastatic brain tumors, which are more common in adults, can arise from various primary neoplasms, with lung, breast, skin, and gastrointestinal tract tumors being common sources (Abolanle et al., 2020). Magnetic Resonance Imaging has become an indispensable tool in the detection and characterization of brain tumors (Byeon et al., 2024). Unlike CT scans, MRI uses powerful magnets and radio waves to create detailed images of the brain and surrounding tissues. One of the significant advantages of MRI over CT is its ability to provide highly detailed and multi-planar images without exposing the patient to ionizing radiation. In comparison to other modalities such as CT scans and PET scans, MRI offers superior soft tissue contrast, making it particularly adept at distinguishing between healthy brain tissue and abnormal growths (Xu and Bai, 2023). This enables clinicians to accurately locate and assess the size, shape, and precise boundaries of tumors, providing crucial information for treatment planning. Dasanayaka et al. (2022b) in their paper discusses brain tumors, highlighting the difference between benign and malignant types, and notes the low survival rates for aggressive forms like Glioblastoma due to challenges in early diagnosis. Moreover, MRI's capability to detect minute changes in tissue composition and vascularity allows for the differentiation of benign and malignant tumors. This is especially important in determining the aggressiveness of the tumor and guiding treatment decisions.

Diagnosing brain tumors using MRI is crucial for treatment planning and patient outcomes (Wang et al., 2021). Various methods have been proposed to enhance the accuracy and efficiency of brain tumor classification. Deep learning techniques, such as DenseNet-ResNet based U-Net frameworks and CRNN models, have shown promising results in extracting features from brain tumor MRI images (Wang C. et al., 2023). Additionally, computer-based techniques utilizing MRI imaging have been developed to detect tumor regions in the brain, categorizing them into healthy brains and those with malignant or benign tumors (Hosseini Saber and Hosseini Saber, 2023). Incorporating advanced imaging techniques like functional MRI, diffusion tensor imaging, perfusion imaging, and spectroscopy aids in differentiating tumor progression from treatment-related changes, enhancing diagnostic capabilities and treatment monitoring (Jordan and Gerstner, 2023). Utilizing deep learning methods like CNN and DWT analysis have shown significant improvements in diagnosing brain tumors, particularly gliomas, with high accuracy and sensitivity (Papadomanolakis et al., 2023). In a recent work by Dasanayaka et al. a Tumor-Analyser is proposed which is a web application that uses interpretable machine learning to classify brain tumors from MRI and whole slide images. It addresses the black-box nature of deep learning models by providing transparent, human-understandable visualizations of the decision-making process (Dasanayaka et al., 2022a). Convolutional Neural Networks (CNNs) in brain tumor analysis from MRI face limitations in explicitly modeling long-term dependencies due to their inherent locality of convolution operations. Thus, CNNs primarily capture local features and have limited ability to model long-range dependencies in images. Brain tumors can have complex spatial relationships and dependencies that may not be effectively captured by CNNs. This can hinder the accurate detection of complex and low-contrast anatomical structures like gliomas in brain MRI (Wang P. et al., 2023). Moreover, CNNs are not inherently invariant to rotations, meaning that their performance can be affected by the orientation of the brain tumors in the MRI scans. This sensitivity to rotations can limit the robustness and reliability of CNN-based classification models. Exploring innovative deep learning architectures like Vision Transformers can offer promising solutions to enhance the accuracy and robustness of brain tumor detection in MRI scans by addressing the limitations of CNNs.

The motivation for Vision Transformers (ViT) in this domain stems from their ability to capture global features and long-range dependencies effectively, which is crucial for precise brain tumor classification (Ferdous et al., 2023). Vision Transformers, by leveraging self-attention mechanisms, excel in extracting global information, thus enhancing the classification accuracy of brain tumors compared to CNNs. Additional, investigating ways to incorporate rotation invariance into ViTs can enhance their robustness and generalization capability. Developing rotation-invariant ViTs can lead to improved classification performance, especially when dealing with brain tumors that may appear in different orientations. The shift toward Vision Transformers in brain tumor analysis aims to overcome the limitations of CNNs in modeling long-range dependencies, rotational invariance and capturing global features for improved accuracy in classification tasks. Thus, the contributions of the proposed method could be summarized as follows:

- To design a rotation invariant ViT (RViT) architecture tailored for the purpose of detecting brain tumors.
- To explore methodologies like rotated patch embedding, whereby rotated iterations of the image are explicitly encoded and analyzed by the RViT.
- To demonstrate the effectiveness and competitiveness of the rotational invariant RViT model in comparison to existing state-of-the-art methods, highlighting its potential for improved brain tumor classification performance.

## 2 Related works

The recent advancements in Vision Transformers (ViTs) have ushered in a paradigm shift in the field of medical imaging, particularly for brain tumor diagnosis and analysis from MRI scans. Unlike traditional convolutional neural network (CNN)-based approaches, ViTs offer novel methodologies that promise to enhance accuracy, efficiency, and interpretability in medical diagnostics.

Pioneering studies have demonstrated the potential of ViTs in this domain. Poornam and Angelina (2024) introduced VITALT, an innovative system that combines ViTs with attention and linear transformation mechanisms for brain tumor detection, showcasing superior performance in classifying tumors from MRI samples and setting a new benchmark for future research. Jahangir et al. (2023) compared the effectiveness of ViTs and CNN-based classifiers, highlighting the unique advantages of ViTs in capturing intricate patterns and features from medical images.

Addressing data scarcity and variance, a key challenge in medical imaging, Haque et al. (2023) proposed a novel approach integrating DCGAN-based data augmentation with ViTs, demonstrating the transformative potential of combining GANs and ViTs for enhanced diagnostic accuracy. Bhimavarapu et al. (2024) developed a system that couples an improved unsupervised clustering approach with a machine learning classifier, aiming to enhance the accuracy of brain tumor detection and categorization.

Further advancements in the field include Natha et al. (2024) multi-model ensemble deep learning approach for automated brain tumor identification, and (Gade et al., 2024) optimized Lite Swin transformer model combined with a barnacle mating optimizer for hyper-parameter tuning, achieving higher classification results and processing efficiency compared to existing transfer learning methods.

Liu et al. (2023) employed an ensemble of ViTs for glioblastoma tumor segmentation, exemplifying the power of combining multiple ViT models to improve segmentation outcomes. Mahmud et al. (2023) proposed a new CNN architecture for brain tumor detection using MRI data and compared its performance to established models like ResNet-50, VGG16, and Inception V3.

In a related work in the field of Alzheimer's Disease diagnosis, Lei B. et al. (2023) proposed FedDAvT, a federated domain adaptation framework using Transformers to diagnose Alzheimer's disease (AD) from multi-site MRI data. They align self-attention maps and use local maximum mean discrepancy to address data heterogeneity while preserving privacy (Lei B. et al., 2023). Similarly, Zuo et al. (2024) develop PALH, a prior-guided adversarial learning model with hypergraphs, to predict abnormal brain connections in AD using fMRI, DTI, and MRI. PALH incorporates anatomical knowledge as prior distribution, employs a pairwise collaborative discriminator, and utilizes a hypergraph perceptual network to fuse multimodal representations. Both studies achieve promising results and provide insights into AD mechanisms (Zuo et al., 2024).

Interdisciplinary applications of ViTs have also emerged, with Babar et al. (2023) unifying genetics and imaging through the classification of MGMT genetic subtypes using ViTs, facilitating personalized treatment plans. Liao et al. (2023) introduced an improved Swin-UNet for brain tumor segmentation, integrating the self-attention mechanism of Swin Transformers with the robust architecture of UNet, pushing the boundaries of medical image segmentation.

Datta and Rohilla (2024) presented a pixel segmentation and detection model for brain tumors, utilizing an aggregation of GAN models with a vision transformer, underscoring the versatility of ViTs in enhancing segmentation precision, especially when combined with advanced data augmentation techniques. Wang P. et al. (2023) offered a comprehensive review on the application of Vision Transformers in multi-modal brain tumor MRI segmentation, serving as a critical resource for understanding the state-of-the-art transformer-based methodologies and their implications for future advancements in medical image segmentation.

These studies collectively present a thorough examination of the current advancements and future potential in employing Vision Transformers for the analysis of brain tumors from MRI scans. Each research contribution introduces distinct viewpoints and methodologies, enhancing our understanding and capabilities in the field of medical imaging diagnostics. The development of a rotationally invariant Vision Transformer (ViT) specifically for the classification of brain tumors in MRI scans is motivated by the variability and critical nature of medical imaging analysis. Conventional imaging techniques often require extensive preprocessing to standardize orientations, which can lead to errors or loss of essential information. A rotationally invariant ViT addresses this issue directly by precisely detecting and classifying brain tumors regardless of their orientation in the scan. This functionality not only improves diagnostic precision but also simplifies the preprocessing workflow, resulting in reduced time and resources required for data preparation. Despite the introduction of various ViT-based methods for brain tumor identification, the exploration of rotational invariance remains unexplored.

## 3 Methodology

ViT represents a significant shift in how neural networks are applied to visual data, diverging from the traditional convolutional neural network (CNN) approach. ViT adopts the transformer architecture, predominantly used for natural language processing tasks. The core idea is to treat an image as a sequence of fixed-size patches, akin to words in a sentence, and apply a transformer model to capture the complex relationships between these patches. This method allows for attention mechanisms to weigh the importance of different image parts dynamically, enabling the model to focus on relevant features for classification or other tasks. The ViT model demonstrated remarkable performance on image classification benchmarks, outperforming state-of-the-art CNNs in certain scenarios, especially when trained on large-scale datasets. This breakthrough underscores the versatility of transformer models and their potential to generalize across different types of data beyond text (Dosovitskiy et al., 2020).

### 3.1 Vision Transformer

The core idea behind ViT is to treat an image as a sequence of fixed-size patches (similar to words in a sentence), apply a transformer to these patches, and use the transformer's output for classification tasks. This method leverages the transformer's capability to capture long-range dependencies, which is beneficial for understanding complex images. The operation of ViT could be break down to following tasks:

#### 3.1.1 Image to patches

An image is split into *N* patches. Each patch is of size *P*×*P*, and the image is of size *H*×*W*×*C*, where *H* and *W* are the height and width, and *C* is the number of channels. Each patch is flattened and linearly projected to a higher dimensional space. Additionally, a learnable embedding is added to each patch embedding.

#### 3.1.2 Position embeddings

Since the transformer architecture does not inherently process sequential data, position embeddings *E*_*pos*_ are added to the patch embeddings *E*_*patch*_ to retain positional information. This is similar to positional encoding in NLP tasks. Hence the embedded patches *ED*_*P*_ could be formulated as in Equation (1).


(1) $$\begin{array}{ll} ED_{P} & =E_{patch}+E_{pos} \end{array}$$

#### 3.1.3 Transformer encoder

The transformer encoder *TF*_*E*_ processes the sequence of embedded patches and returns processed output *TF*_*O*_. It consists of multiple layers, each with multi-head self-attention and feed-forward networks, allowing the model to capture complex relations between patches as given in Equation (2).


(2) $$\begin{array}{l} TF_{O}=TF_{E}\left(ED_{P}\right) \end{array}$$

#### 3.1.4 Classification head

The output of the transformer encoder is passed to a classification head, typically a linear layer, to predict the class labels. Often, the output corresponding to a special class token added to the sequence is used for classification as given in Equation (3).


(3) $$\begin{array}{ll} y & =\text{Softmax}\left(\text{W}\cdot {\text{h}}_{\left[\text{CLS}\right]}\right) \end{array}$$

Here, **W** represents the weights of the linear layer, and **h**_[CLS]_ represents the output of the transformer encoder corresponding to the class token.

### 3.2 Proposed rotation invariant ViT

In computer vision, the performance of a model can be significantly affected by variations in the input data, such as changes in orientation. Most convolutional neural networks (CNNs) and Vision Transformers (ViTs) are not inherently rotation-invariant, meaning that if an image is rotated, the model may not recognize the objects in the image as effectively as it does when they are in their original orientation (Lei T. et al., 2023). The motivation for rotating images and then performing patch embedding for rotational invariance is to make the model more robust to such rotations without the need for extensive data augmentation or more complex model architectures.

**Figure 1:**
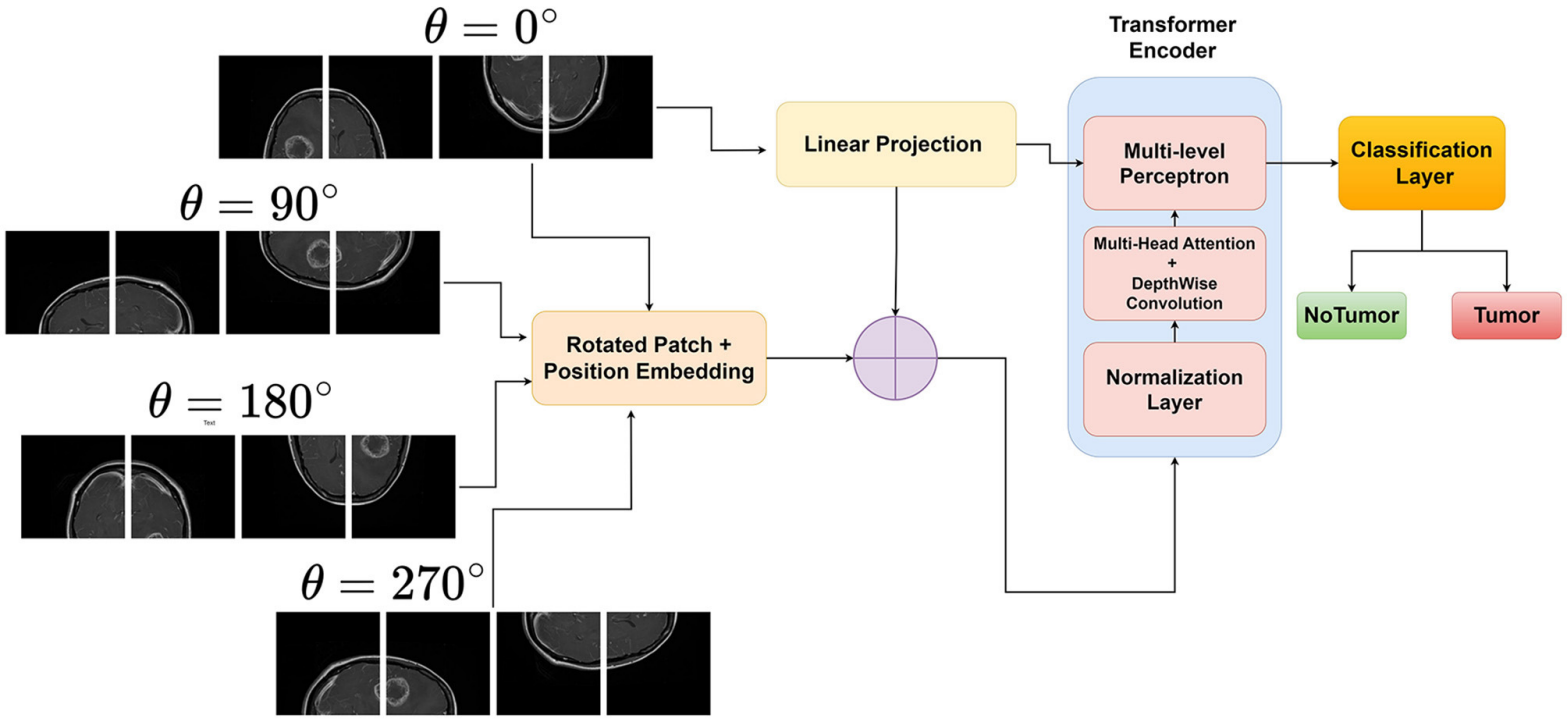
Proposed rotation invariant Vision Transformer for brain tumor classification from MRI.

Rotational invariance is a desirable property for many computer vision tasks, as objects in images can appear in different orientations without changing their semantic meaning. However, traditional Vision Transformers (ViTs) are not inherently invariant to rotations, which can limit their performance and generalization ability when dealing with rotated objects (Heo et al., 2024; Su et al., 2024). This limitation motivates the development of rotational invariant ViTs. By explicitly encoding rotated image patches and then performing input enbedding, ViTs can learn to be invariant to rotations of the input image. This is achieved by generating rotated versions of the images and assigning them unique rotation embeddings. Figure 1 illustrates the novel approach of the proposed rotation invariant ViT(RViT) for brain tumor classification. The components of the block diagram are explained further in the following sections.

#### 3.2.1 Generating rotated patches

Let *X*∈ℝ^*H*×*W*×*C*^ be the input image, where *H*, *W*, and *C* denote the height, width, and number of channels, respectively. The image is divided into a grid of fixed-size patches $P_{i}\in {\mathbb{R}}^{N\times \left(P^{2}\cdot C\right)}$, where *N* is the number of patches, *P* is the patch size, and *P*^2^·*C* is the flattened patch dimension.

Furthermore, for the same image *X*, we generate its rotated versions *X*^(*j*)^, where *j*∈{0, 1, 2, 3, …} corresponds to the different rotation angles θ. The rotated image tensor *X*^(*j*)^ can be represented as *X*^(*j*)^∈ℝ^*H*×*W*×*C*^ after rotation, and then we extract and flatten patches as before, resulting in $R_{i}^{\left(j\right)}\in {\mathbb{R}}^{N\times \left(P^{2}\cdot C\right)}$.

#### 3.2.2 Patch embedding

Each rotated patch $R_{i}^{\left(j\right)}$ of the original input *X*^(*j*)^ is linearly projected using an embedding matrix $E\in {\mathbb{R}}^{(P^{2}\cdot C)\times D}$, where *D* is the embedding dimension.

The patch embedding $Z_{i}^{\left(j\right)}$ for each rotation *j* is obtained as given in Equation (4):


(4) $$\begin{array}{l} Z_{i}^{\left(j\right)}=R_{i}^{\left(j\right)}E \end{array}$$

#### 3.2.3 Averaging the embeddings

For rotational invariance, we take the average of the embeddings from the original and rotated patches. If we have *k* rotations, the final embedding for a patch is given by Equation (5):


(5) $$\begin{array}{l} Z_{i}=\frac{1}{k}\overset{k-1}{\underset{j=0}{\sum }}Z_{i}^{\left(j\right)} \end{array}$$

This averaged embedding *Z*_*i*_ is then passed through the subsequent layers of the Vision Transformer for further processing.

#### 3.2.4 Forward pass through transformer encoder

The sequence of embedded patches is passed through *L* layers of the transformer encoder as in Equation (6):


(6) $$\begin{array}{l} TF_{O}=TF_{E}\left(Z_{i}\right) \end{array}$$

Each Transformer Encoder Layer typically consists of multi-head self-attention and feed-forward neural networks. The output of the last transformer encoder layer is used by the classification head as shown in Equations (2) and (3):

Generally, the class token is typically the first token in the sequence after the last encoder layer, which is used as the representation for classification. The Classifier Head can be a simple linear layer or a more complex neural network. This results in the final output *y*, which is the class prediction for the input image *X*. The rotational invariance technique used in the RViT model is also outlined in Algorithm 1.

**Algorithm 1:**
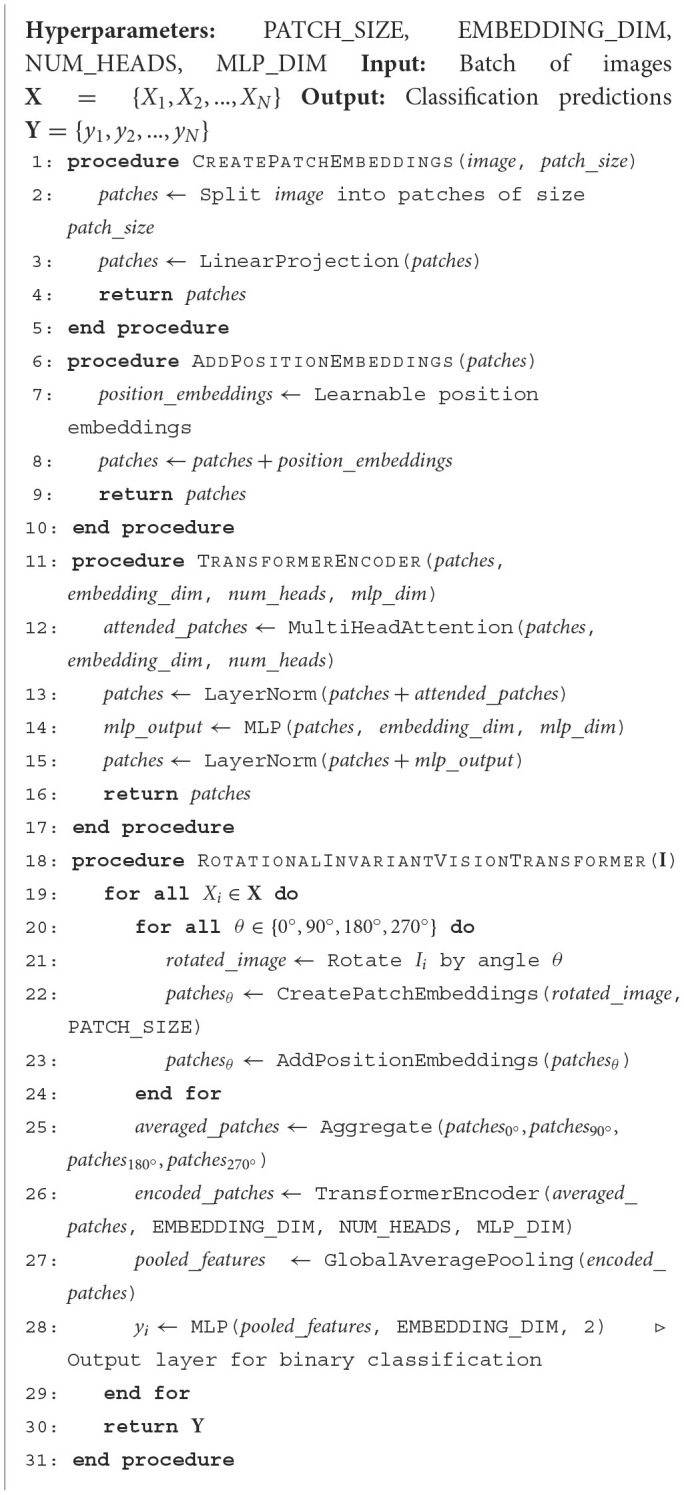
Rotation invariant Vision Transformer.

## 4 Experimental analysis

For the experimental validation, we utilized the Brain Tumor MRI Dataset available on Kaggle at https://www.kaggle.com/datasets/masoudnickparvar/brain-tumor-mri-dataset. This dataset contains MRI scans of brain tumors, specifically focusing on glioma tumors and non-tumor cases. From the dataset, we selected the glioma and non-tumor MRI images for our analysis. The training set consists of 1,321 glioma images and 1,595 non-tumor images as provided in the Kaggle dataset, providing a substantial amount of data for training our models (Nickparvar, 2021). For the testing set, we allocated 300 glioma images and 405 non-tumor images to evaluate the performance and generalization ability of the trained models. The training data was directly sourced from the dataset and split into an 80% training set and a 20% validation set for model training and evaluation. The testing images were used as provided in the dataset, ensuring that the test cases represent the real-world scenario. This approach maintains a balanced representation of glioma and non-tumor images in both the training and testing phases, facilitating robust model training and evaluation.

By utilizing the above mentioned dataset, we aim to develop and validate our rotation invariant Vision Transformer (ViT) model for accurate brain tumor classification, comparing its performance against state-of-the-art deep learning architectures. The dataset's diverse collection of glioma and non-tumor MRI scans serves as a reliable benchmark for assessing the effectiveness of our proposed approach in real-world clinical scenarios. The technical implementation of the RViT and its variant models utilized an RTX4000 GPU with 8 GB VRAM, 30 GB RAM, and an 8-core CPU. The model was developed using PyTorch version 1.12, a deep learning framework offering diverse functions and libraries for model training and evaluation.

In the experimental analysis, our Rotation invariant Vision Transformer (RViT) and a baseline Vision Transformer (Base-ViT) were used for brain tumor detection. Both models were configured with distinct hyperparameters as outlined in Tables 1, 2. For RViT and Base-ViT, we set a standard patch size of 16. However, RViT had a depth of 10 while Base-ViT had a depth of 12. Notably, the MLP size for Base-ViT was set to 1,024, compared to RViT's 480. The parameters of Base-ViT are selected based on published literature (Dosovitskiy et al., 2020). Each model was trained using Adam optimizer, with an identical learning rate of 0.001 and weight decay of 0.01.

**Table 1:** Hyperparameters of RViT.

**Parameter**	**Description**
Image size	224 × 224
Patch Size	16
Patch embedding dimension	142
Depth	10
Number of heads	10
MLP size	480
Embedding integration	Average
Batch size	32
Attention dropout	0.1
Optimizer	Adam
Weight decay	0.01
Learning rate	0.001

**Table 2:** Hyperparameters of the Base-ViT.

**Parameter**	**Description**
Image size	224 × 224
Patch size	16
Patch embedding dimension	768
Depth	12
Number of heads	12
MLP size	1,024
Batch size	32
Attention dropout	0.1
Optimizer	Adam
Weight decay	0.01
Learning rate	0.001

**Figure 2:**
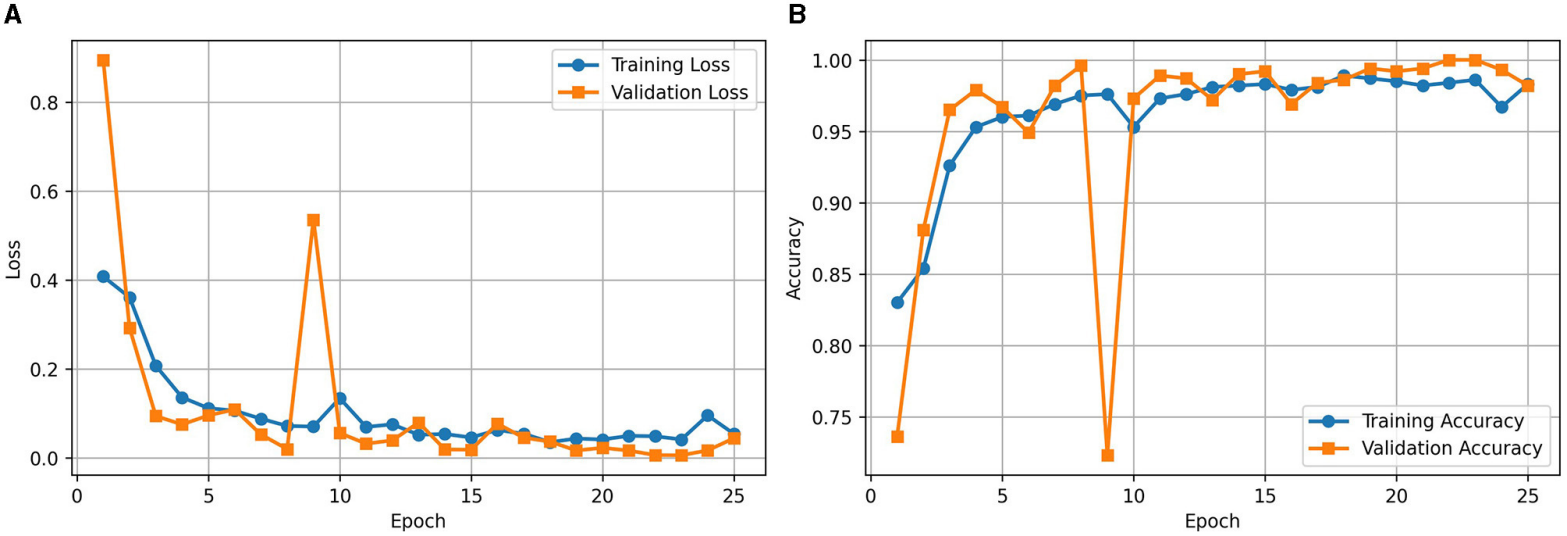
**(A)** Loss and **(B)** accuracy curves for the proposed rotation invariant ViT (RViT).

Figure 2A illustrates the loss curve for the proposed approach of using RViT. We could observe that training and validation loss showing a decreasing trend over epochs indicates that the model is learning effectively. Similarly, Figure 2B shows the accuracy curve of the RViT showing an increasing trend over epochs indicating that the model's performance is improving. At the end of the training and validation phase the model is stored for determining the classification performance of the tumor detection based on test dataset.

**Figure 3:**
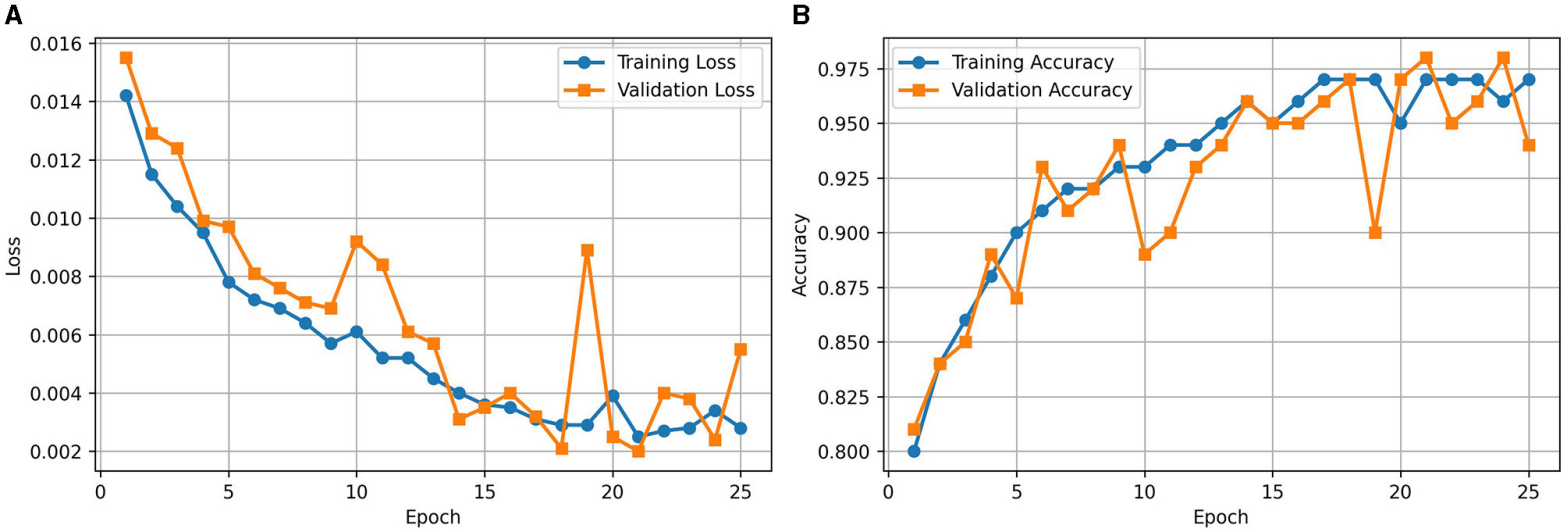
**(A)** Loss and **(B)** accuracy curves for Base-ViT.

During the training of the Base-ViT model as illustrated in Figure 3, both the loss and accuracy demonstrated improvement over 25 epochs, with the training loss decreasing significantly and the training accuracy reaching a steady value above 93%. The validation loss and accuracy fluctuated but generally followed the training trends, indicating good generalization without overfitting.

### 4.1 Ablation study

Our investigation of the RViT model through ablation studies plays a crucial role in elucidating the importance of its architectural elements. At the outset, we eliminated the rotated patch embedding scheme, a key feature that enables the model to address rotational variability in imaging data. This methodology usually consists of partitioning the image into patches and implementing rotations to account for the diversity in image orientation, a critical process for precise classification endeavors in medical imaging.

Furthermore, we explored the impact of omitting depth-wise convolutional layers from the architecture. Depth-wise convolutions are an efficient variant of the standard convolution that processes each input channel independently, thus reducing computational complexity and preserving spatial hierarchies. Their inclusion in Vision Transformers (ViTs) like ours is not standard but can provide localized spatial filtering, which is beneficial for tasks that require detailed spatial understanding, such as detecting complex structures in MRI scans.

**Figure 4:**
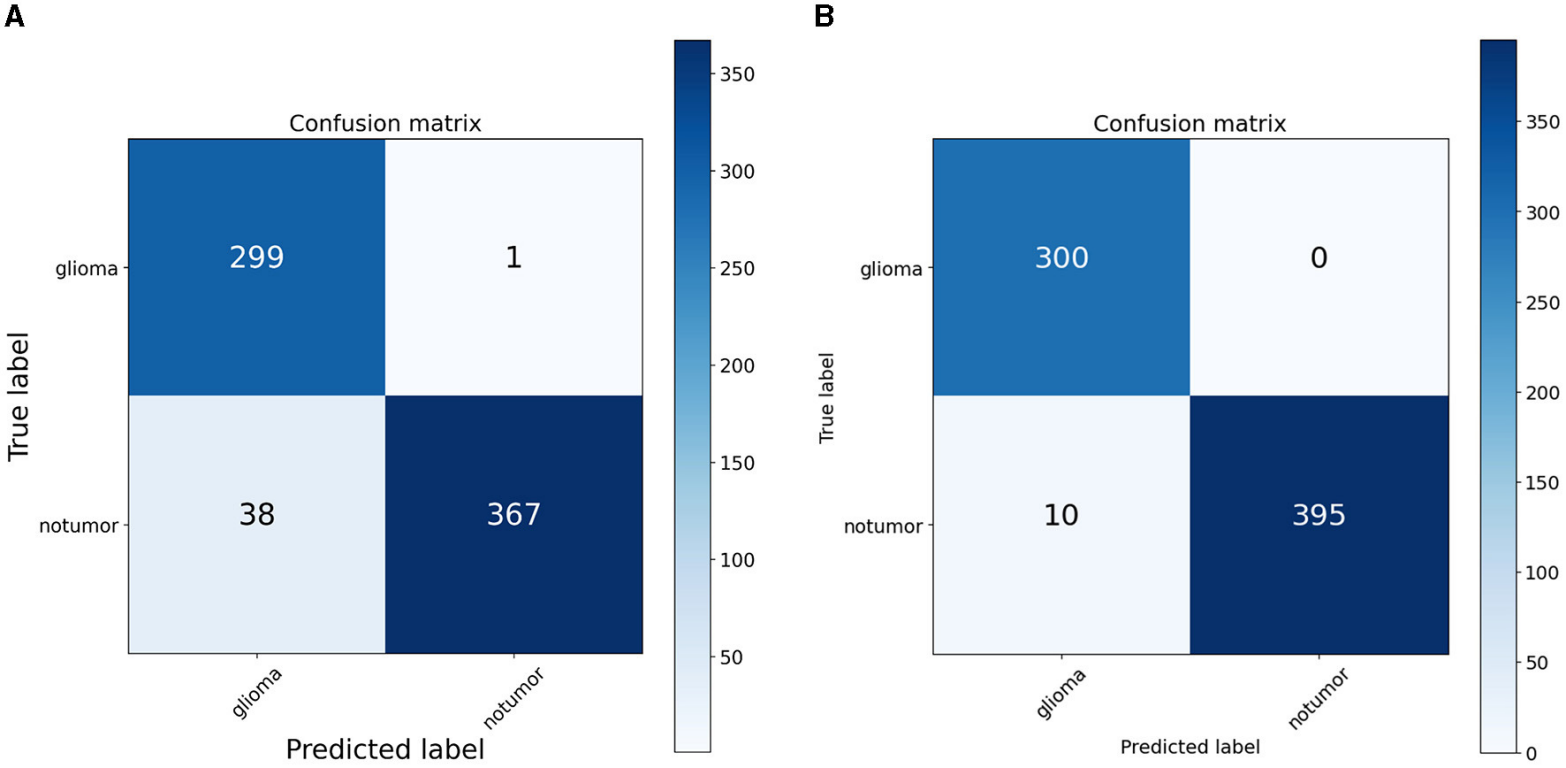
Confusion matrix. **(A)** ViT. **(B)** Proposed RViT.

**Figure 5:**
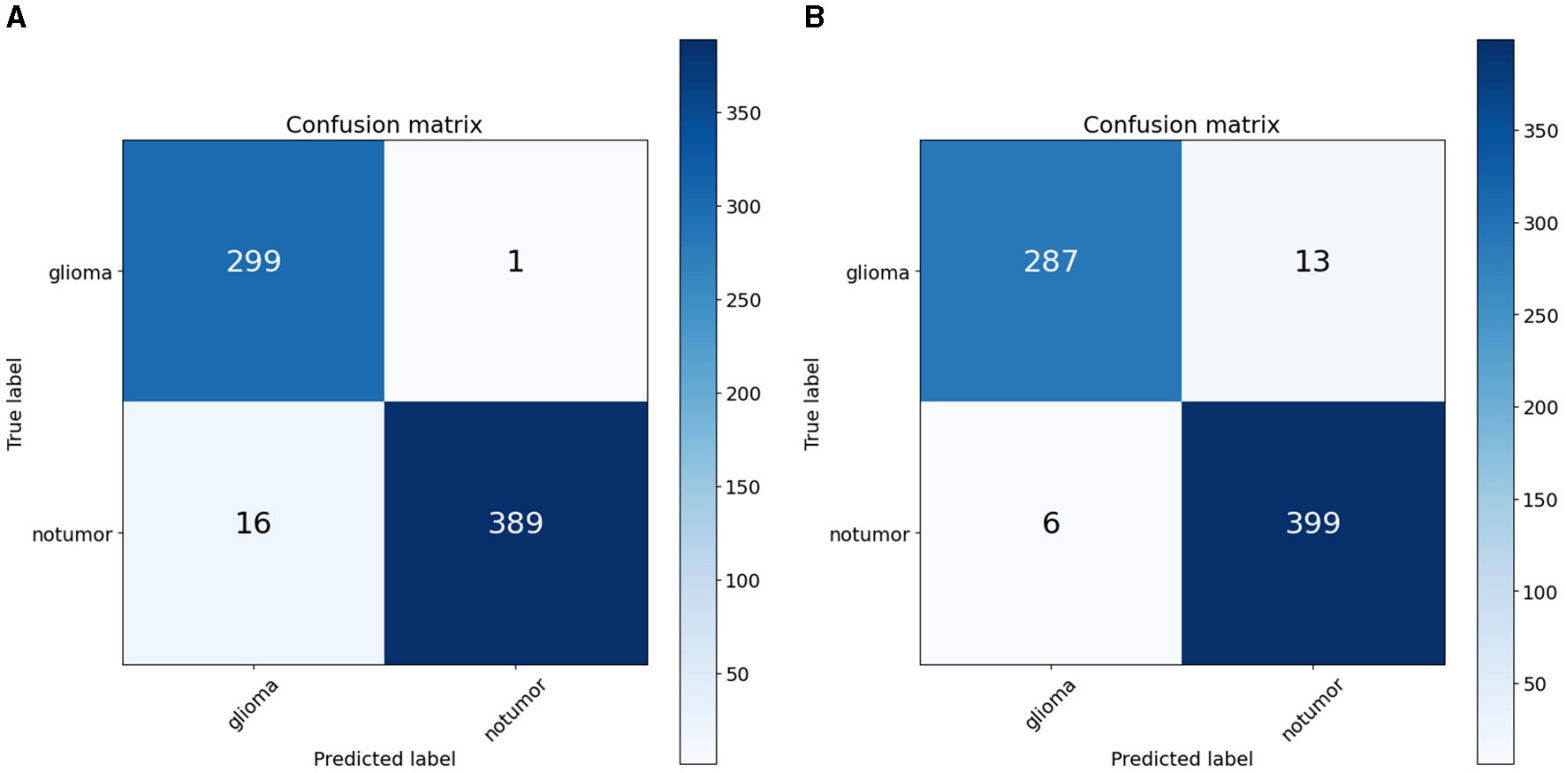
Confusion matrix. **(A)** RViT—variant1. **(B)** RViT—variant2.

To quantify the effects of these modifications, we conducted experiments across all variants of the RViT model. The results of these experiments were captured in confusion matrices, displayed in Figure 4 for the baseline ViT and our proposed RViT model, and Figure 5 for the two variants of RViT. The matrices reveal the models' performance in distinguishing glioma from non-tumor MRI scans. Figure 4B shows the superior performance of the proposed RViT, with perfect identification of glioma cases and a high true negative rate. In contrast, Figure 5 illustrates the outcomes for the RViT variants, highlighting the decrement in performance upon removing rotated patches and depth-wise convolutions, as evidenced by increased false negatives and false positives, respectively. These findings underscore the critical role of rotational invariance and depth-wise convolutions in our RViT model's ability to accurately classify brain tumors.

The evaluation of the deep learning models as presented in Tables 3, 4 showcases the performance and efficiency trade-offs between the Base-ViT, the full RViT model, and its ablated variants. The RViT outperforms the base Vision Transformer in terms of accuracy (ACC), achieving a 0.986 score compared to ViT's 0.944. It also maintains high sensitivity and specificity, although there is only a slight difference in sensitivity when compared to the baseline ViT. This trade-off comes with significant gains in model efficiency, as seen in Table 4, where the RViT has considerably fewer parameters (7,527,612) compared to the ViT's 38,558,978, without a significant compromise on performance metrics.

**Table 3:** Performance metrics of the deep learning models studied here that includes base-ViT, proposed RViT and its variants.

**Method**	**Sensitivity**	**Specificity**	**F1-score**	**MCC**	**ACC**
ViT	0.996	0.906	0.938	0.893	0.944
RViT	1.0	0.975	0.984	0.972	0.986
RViT_Variant1	0.996	0.960	0.972	0.951	0.975
RViT_Variant2	0.956	0.985	0.968	0.944	0.973

**Table 4:** Parameter size of the deep learning models evaluated here.

**Name**	**# Parameters**	**Training time (sec) epochs = 25**
ViT	38,558,978	5,802
RViT	7,527,612	2,055
RViTVariant1	7,527,612	1,842
RViT_Variant2	5,826,562	1,687

The ablation studies highlighted in RViT_Variant1 (No Rotated Patch Embedding) and RViT_Variant2 (No Depth-wise Convolution) show a marginal decrease in performance metrics, including F1-score and Matthew's Correlation Coefficient (MCC), when depth-wise convolutions and rotational patch embedding are removed. Notably, RViT achieves a higher ACC than the baseline ViT, RViT_Variant1 and RViT_Variant2 demonstrates that rotational patch embedding contributes to performance.

These findings are significant for clinical applications where both accuracy and computational efficiency are crucial. The reduced parameter count and shorter training time of the RViT and its variants as shown in Table 4, as compared to the base ViT, underscore the potential of these models for scalable and efficient medical image analysis. The RViT model not only excels in performance by offering high accuracy but also requires fewer parameters and less training time compared to the baseline ViT model experimented here.

**Figure 6:**
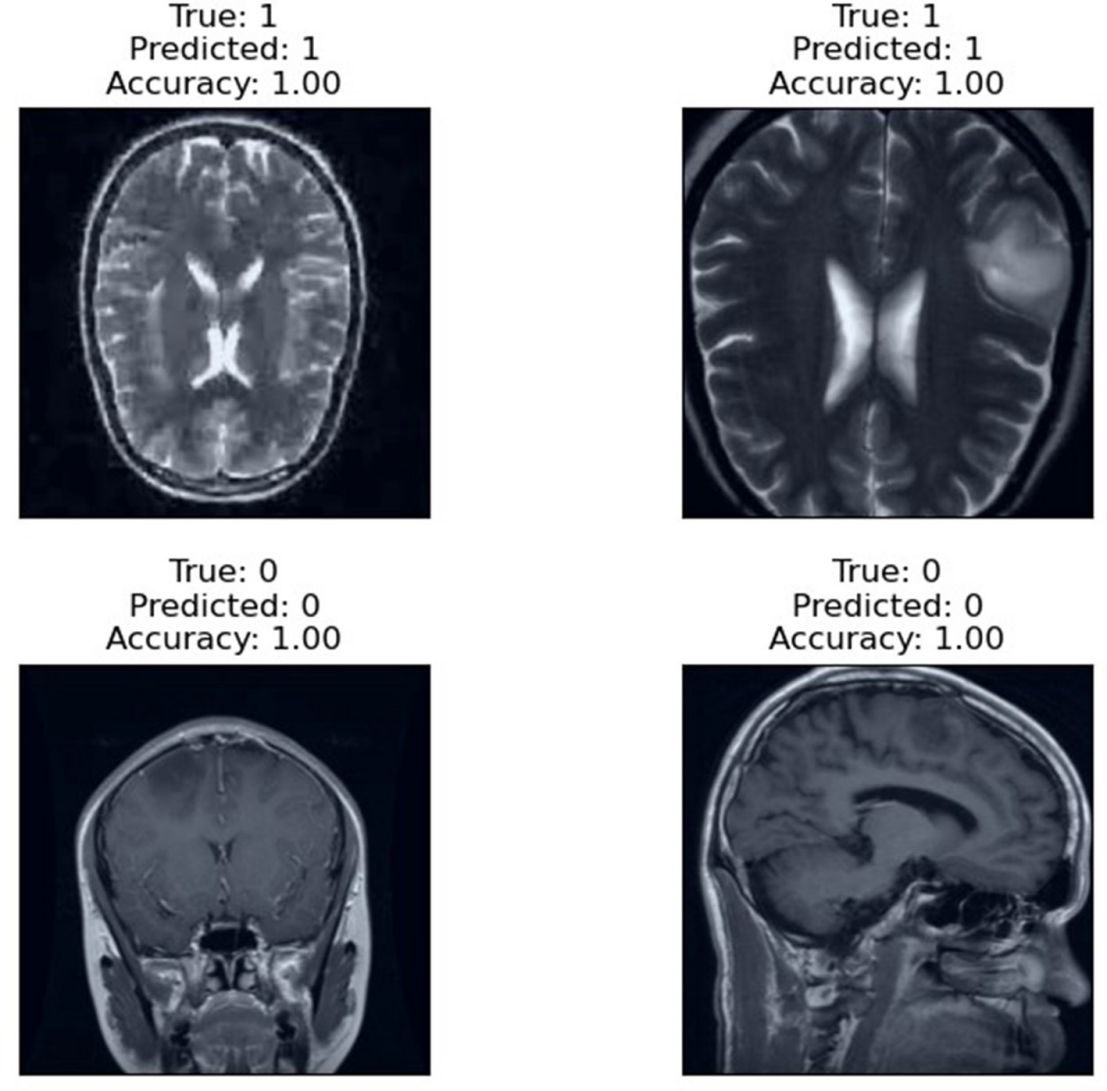
Prediction result of the proposed RViT.

The prediction results of the proposed RViT model, as depicted in Figure 6, are a testament to its robustness. The model accurately predicts the presence or absence of glioma in MRI images with an accuracy score of 1.00. The top row of the figure presents cases with glioma (True: 1), and the RViT model correctly identifies them (Predicted: 1), showcasing its effectiveness in recognizing complex tumor patterns. Similarly, the bottom row demonstrates the model's precision in identifying non-tumor images (True: 0) with perfect accuracy (Predicted: 0). These results underline the model's capability to handle various imaging rotations, ensuring high reliability in detecting brain tumors'a crucial requirement for aiding diagnostic procedures in healthcare.

## 5 Discussion

The experimental analysis done in this research work suggests that, the Rotation Invariant Vision Transformer (RViT) model is effective in brain tumor classification, outperforming several state-of-the-art methodologies. Specifically, the RViT's incorporation of rotational patch embeddings permits adept handling of rotational variations in MRI scans, a notable limitation in conventional Vision Transformers. Comparative analysis reveals the RViT's precision; for instance, it achieves an accuracy (ACC) of 0.986 and perfect Precision (PREC) for non-tumor identification, surpassing other approaches like the Lite Swin transformer and Fuzzy c-Means+Extreme ML approaches, which exhibit marginally lower precision in the same tasks, according to the Table 5 summary (Bhimavarapu et al., 2024; Gade et al., 2024). This suggests RViT's pronounced ability to accurately distinguish between glioma and non-tumor instances, validated by the performance scores from our experimental results.

**Table 5:** Comparative analysis of the proposed method with some of the state-of-the -art methods for tumor classification based on Kaggle dataset.

**Study**	**Method**	**Class**	**Performance scores**			
			**ACC**	**SENS**	**PREC**	**F1**
VSR Gade et al. (2024)	Lite Swin transformer	No tumor	0.980	0.962	0.921	–
		Glioma	0.968	0.943	0.939	–
Bhimavarapu et al. (2024)	Fuzzy C-Means + Extreme ML	No tumor	0.994	0.996	0.998	–
		Glioma	0.992	0.999	0.997	–
Mahmud et al. (2023)	Pre-trained CNN models	No tumor	0.935	0.956	0.944	–
		Glioma	0.931	0.956	0.946	–
Natha et al. (2024)	SETL_BMRI	No tumor	0.987	0.990	1.000	0.990
		Glioma	0.987	0.970	1.000	0.990
Proposed	RViT	No tumor	0.986	0.975	1.000	0.988
		Glioma	0.986	1.0	0.968	0.984

Moreover, when scrutinizing the model's sensitivity (SENS), the RViT model impeccably identifies glioma instances (SENS = 1.0), as indicated in the comparison table, reflecting its acumen in detecting true positive cases without fail. The proficiency of RViT is further substantiated by the F1 scores it garners, which remain comparable to its counterparts. Such efficacy is a testament to RViT's specialized architecture, adeptly engineered to navigate the intricacies of brain tumor MRIs.

**Figure 7:**
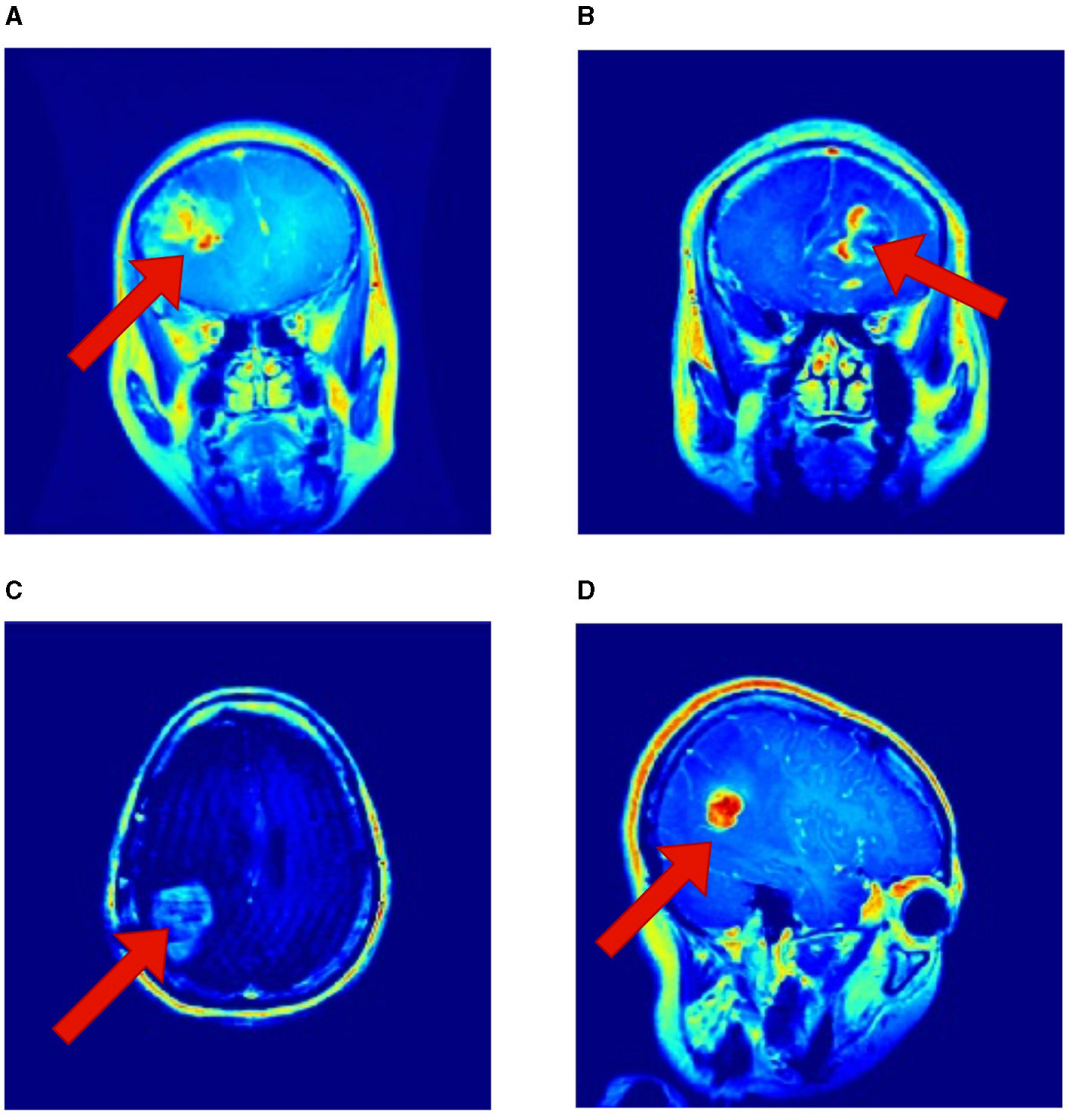
Interpretability of the proposed RViT model based on GradCAM method for a sample of test images provided in the Kaggle dataset. **(A–D)** shows activations maps of the sample images (Selvaraju et al., 2019).

It is essential to recognize that the literature employing the same Kaggle dataset has underscored the robustness of RViT. When juxtaposed with related works, RViT's optimized model unequivocally demonstrates better classification results and processing efficiency, thus, underscoring its superiority. RViT's combination of Rotated Patch embedding and Depth-wise convolutions are pivotal for its high accuracy and minimal false predictions, as reflected in the confusion matrix for the baseline ViT and proposed RViT shown in Figure 4. The importance of these architectural components is further highlighted by the confusion matrices in Figure 5, which illustrate the decrement in performance upon removing rotated patches and depth-wise convolutions, respectively. Furthermore, the interpretability of the RViT model is analyzed using GradCAM visualizations presented in Figure 7. These visualizations reveal that the tumor regions exhibit higher activations, indicated by the arrows, suggesting that the proposed model effectively learns tumor regions based on their textures. However, the visualizations also highlight regions attributed to the model's decisions due to similar intensity levels as tumor regions, despite not containing actual tumors. This interpretability analysis enhances the transparency and trustworthiness of the proposed approach while also identifying potential areas for further improvement in the model's decision-making process.

The major limitations are, the computational intensity of the rotational embeddings in the RViT model is not trivial, though its accuracy is without question at the forefront. The balance between computational demand and the precision of the model is critical, particularly when considering the extensive dataset needed to maximize RViT's proficiency. While this comprehensive dataset fortifies the model's robustness and its ability to generalize, it also hints at the untapped potential of RViT, as the full breadth of its capabilities has yet to be fully explored. This aspect becomes critical when envisioning RViT's deployment in clinical environments where it must interpret a vast spectrum of MRI scans effectively.

The efficiency of RViT is anchored not only in its architectural design but also in the thorough experimentation to which it is subjected. The confluence of these factors culminates in the model's adeptness at classification tasks, as the data tables suggest, pointing to the transformative promise of RViT in the realms of brain tumor detection and classification within the medical field.

However, a pertinent limitation is the model's focus on binary classification, whereas other studies in the field often tackle multiclass scenarios, presenting a more nuanced challenge (Mahmud et al., 2023; Natha et al., 2024). Additionally, the incorporation of rotational patch embedding introduces an extra layer of complexity, but this does not translate to an increase in hyperparameters due to the rotational operations. It's important to note that the RViT model currently contemplates only four rotational orientations. This represents a limited scope as real-world medical scenarios may encounter a wider range of orientations, which necessitates further investigation to ensure the model's applicability across more varied diagnostic situations.

## 6 Conclusion

This research introduces the Rotation Invariant Vision Transformer (RViT) as a powerful model for brain tumor classification from MRI scans. Our model addresses the rotational variance in brain tumor imaging, a significant challenge for traditional deep learning models. The RViT's incorporation of rotational patch embeddings allows it to detect and classify brain tumors with high sensitivity and specificity, achieving an overall accuracy that surpasses the base Vision Transformer model and current state-of-the-art methods. Through experimental validation using the Brain Tumor MRI Dataset from Kaggle, the RViT demonstrated its robustness, outperforming other techniques with impressive Sensitivity and Specificity. It is particularly adept at handling the complex spatial relationships and dependencies characteristic of gliomas, as evidenced by its perfect classification results.

However, the study acknowledges limitations, notably the binary nature of the classification, while many practical applications may require multiclass capabilities. Moreover, the RViT considers only a limited number of rotational orientations, suggesting the need for further research into models that can handle an expanded range of tumor appearances. The rotational patch embedding introduces additional complexity but does not lead to an increase in the model's hyperparameters, maintaining computational efficiency. The research also points to the need for a larger, more diverse dataset to enhance the model's robustness and generalization ability. While the RViT's current performance is promising, its full potential is yet to be tapped with a more extensive dataset. This expansion would not only bolster the model's diagnostic accuracy but also its applicability to real-world clinical settings where the variance in tumor presentation is vast.

The RViT represents a significant step forward in the application of Vision Transformers to medical diagnostics. Its design, combining the power of deep learning with an innovative approach to rotational invariance, has the potential to streamline brain tumor detection and classification, ultimately leading to better patient outcomes. Future work will look to address these limitations by expanding the number of rotational orientations considered, exploring multiclass classification scenarios, and testing the model on a broader dataset. Additionally, there is potential in exploring how the RViT framework could be adapted or extended to other medical imaging modalities and diagnostic tasks.

The findings of this study contribute to the ongoing evolution of AI in medical imaging and highlight the importance of specialized model architectures like RViT in addressing the unique challenges presented by complex imaging data. With continued research and development, models like RViT could soon become a standard tool in clinical diagnostics, aiding physicians in the accurate and efficient diagnosis of brain tumors and potentially other conditions.

## Data availability statement

Publicly available datasets were analyzed in this study. This data can be found here: https://www.kaggle.com/datasets/masoudnickparvar/brain-tumor-mri-dataset.

## Ethics statement

Ethical approval was not required for the study involving humans in accordance with the local legislation and institutional requirements. Written informed consent to participate in this study was not required from the participants or the participants' legal guardians/next of kin in accordance with the national legislation and the institutional requirements.

## Author contributions

PTK: Conceptualization, Writing – original draft. PK: Writing – review & editing, Software, Validation, Visualization. MK: Writing – review & editing, Data curation, Formal analysis. DG: Data curation, Writing – review & editing. AN: Resources, Software, Writing – review & editing. TK: Funding acquisition, Supervision, Writing – review & editing.
